# A rare presentation of mycotic cerebral aneurysm, subarachnoid haemorrhage, and mitral valve aneurysm in left-sided lnfective endocarditis: a case report and literature review

**DOI:** 10.1093/ehjcr/ytad567

**Published:** 2023-11-21

**Authors:** Ahmed A M Abbas, Pamela F Brown, Ralph W White

**Affiliations:** Department of Surgery, University of Warith Al-Anbiyaa, Iraq-Holy Karbala / Baghdad - Karbala Road (7km from Downtown), Iraq; Cardiothoracic Surgery Department, James Cook University Hospital, Marton Rd, Middlesbrough TS4 3BW, UK; Cardiothoracic Surgery Department, James Cook University Hospital, Marton Rd, Middlesbrough TS4 3BW, UK

**Keywords:** Mitral valve aneurysm, Left-sided infective endocarditis, Mycotic cerebral aneurysm, Subarachnoid haemorrhage, Case report

## Abstract

**Background:**

Infective endocarditis (IE) can present as a syndromic-like condition with multisystem involvement; this can make early diagnosis particularly challenging. Rarely, left-sided IE can lead to mitral valve aneurysm formation. Showering of septic emboli to the cerebral circulation may result in a mycotic aneurysm that can rupture, leading to haemorrhagic stroke, as in this case.

**Case summary:**

A 28-year-old male presented with a triad of *subarachnoid haemorrhage* (SAH) from mycotic cerebral aneurysm rupture, *left-sided aortic and mitral valve IE* causing severe regurgitation and aorto-mitral curtain fistula and *mitral valve aneurysm formation*. The SAH was the main initial presentation and was immediately treated with coiling by an interventional radiologist. However, the patient later developed heart failure due to severe aortic and mitral valve regurgitation that led to the diagnosis of IE. The patient underwent aortic and mitral valve replacements procedure10 days after SAH presentation. He then recovered satisfactorily from the operationa and successfully discharged home after completeing his course of intravenous antibiotics.

**Discussion:**

In this article, we shed some light on this unusual syndromic presentation, elaborate on the underlying mechanism, the ultimate importance of clinical examination, pitfalls in diagnosis, the important role of the heart team in IE, and finally the timing of surgery after SAH.

**Learning points**

- This case presents a unique triad of left-sided endocarditis, mitral valve aneurysm, and cerebral mycotic aneurysm in one presentation.
- This case scenario highlights several learning outcomes including the importance of holistic clinical examination of the cardiovascular system in a patient presenting with haemorrhagic stroke; a high index of suspicion of infective endocarditis is needed in cases of cerebral aneurysm with no risk factors and presence of non-specific history of lethargy or flu-like illness.
- Vasopressors are best avoided in such cases in the context of aortic regurgitation due to the risk of acute decompensation.
- This case shows that cardiac surgery seems to be safe as early as 10 days after subarachnoid haemorrhage provided that the aneurysm is coiled.

## Introduction

Infective endocarditis (IE) is one of the great masqueraders in modern medicine due to diverse and complex multisystem involvement that can lead to delayed diagnosis. In this case report, we highlight an unusual case of IE presenting with subarachnoid haemorrhage (SAH) due to ruptured cerebral mycotic aneurysm with concomitant aortic and mitral valve involvement and complicated by mitral valve aneurysm. The link between IE and mycotic aneurysm is well documented.^1^ The term mycotic was initially confined to *Salmonella typhi*, but modern medicine has used it liberally for any microorganism.^1^ Currently, the term infectious intracranial aneurysm is more accurate and acceptable to describe any aneurysm with infectious origin.^2^

Mitral valve aneurysm is frequently reported in the literature and is well linked to aortic valve endocarditis. Involvement of both anterior and posterior leaflets is reported, but the anterior one is the most common.^3^ What makes this case unique and noteworthy is the rare simultaneous presentation of IE with cerebral mycotic aneurysm causing SAH and mitral valve aneurysm, along with the lessons learned from managing this case.

## Summary figure

**Table:** 

2 Months prior admission	Lethargy associated with 15 kg weight loss over the same period, exertional palpitations, recurrent collapses with no loss of consciousness, night sweats, and recent diagnosis of anaemia
Day 1 of admission	Severe frontal headache and photophobia
	Computed tomography (CT) angiogram with cerebral embolization. The aneurysm in the middle cerebral artery branch, including the parent vessel, was occluded using multiple target detachable coils (Stryker, USA).
Day 2	Sent to recover in the ward under fluid and vasopressor (argipressin) therapy to keep his blood pressure between 160 and 180 mmHg
Day 3	Developed high oxygen requirement with a clinical and radiological picture of pulmonary oedema and was therefore admitted to the critical care unit where he was stabilized using high-flow oxygen therapy.
Day 6	Trans-thoracic echo revealed severe aortic regurgitation (AR), a bicuspid aortic valve, anterior mitral valve leaflet aneurysm, and severe mitral regurgitation. Multiple site blood cultures grew *Streptococcus sanguinis*, and policy-guided intravenous (IV) antibiotic therapy commenced.
Day 8	Trans-oesophageal echo revealed aortic valve vegetation, mitral valve aneurysm, and anterior mitral valve leaflet perforation with suspicion of root abscess at the level of aorto-mitral curtain.
Day 10	Mechanical aortic and mitral valve replacement with patch reconstruction of the aorto-mitral curtain
Day 31	The patient was discharged home well to finish the 6-week antibiotic course.

## Case report

A 28-year-old fit and well non-smoking male factory worker with no history of alcohol or IV drug use presented to the emergency department with 2-day history of severe frontal headache and photophobia. He reported lethargy for 2 months associated with 15 kg weight loss over the same period, exertional palpitations, night sweats, and recurrent collapses with no loss of consciousness and had a recent diagnosis of anaemia. During this time, he was managed by his general practitioner, and his flu-like symptoms were attributed to COVID-19. He had a background of antibiotics for a dental infection 7 months earlier and COVID-19 infection 2 months prior to that.

**Figure 1:**
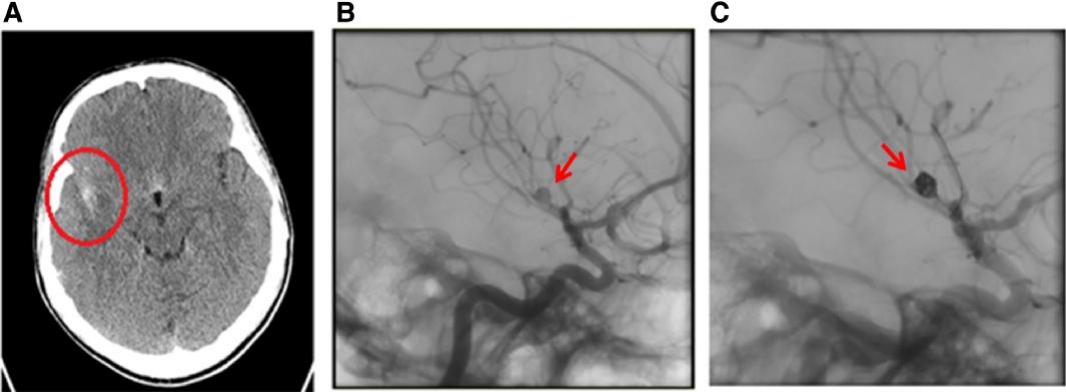
(*A*) Computed tomography brain shows acute subarachnoid haemorrhage with blood mainly seen in the right Sylvian fissure. (*B*) A 4 mm irregular surface aneurysm (arrow) with wide neck incorporating the segment of the artery arising from the proximal trunk of the superior M2 bifurcation of the right middle cerebral artery. This appears to be an acutely ruptured dissecting aneurysm. (*C*) The mycotic aneurysm post-coiling (arrow).

On admission, he had photophobia but no neurological deficit and was apyrexial with normal haemodynamics. He was found to be anaemic (haemoglobin 103 g/L) with elevated inflammatory markers (white cell count 14.8 × 10^9^/L and C-reactive protein was 35 mg/L). The initial differential diagnosis included intracranial haemorrhage and intracranial space-occupying lesion. An urgent cranial CT was performed, and the findings are shown and described in *Figure 1A* and *B*.

**Figure 2:**
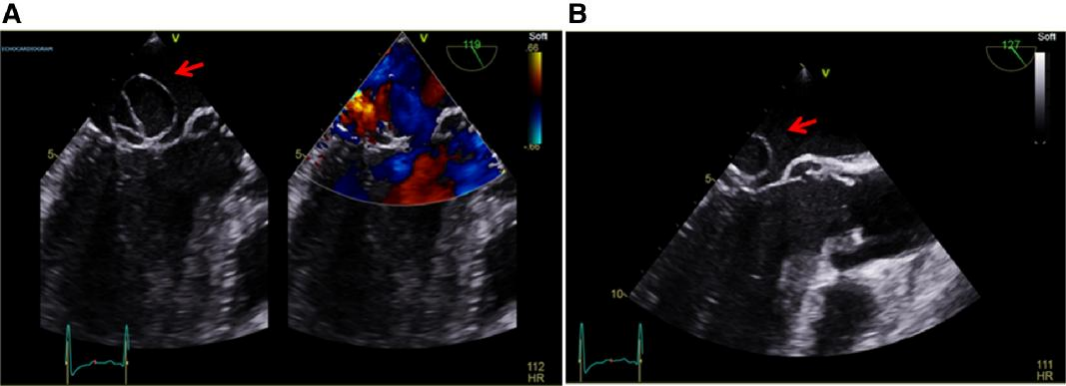
A long-axis trans-oesophageal echocardiogram showing the anterior mitral leaflet aneurysm (see the arrows) and eccentric Doppler jet.

The patient underwent a cerebral angiogram that diagnosed cerebral mycotic aneurysm at the same time. The aneurysm was successfully treated immediately by an interventional radiology with endovascular coiling (*Figure 1C*), and the patient was sent to recover with a target systolic blood pressure control of 160–180 mmHg using fluid and vasopressor (argipressin). The next day, he developed a high oxygen requirement with a clinical and radiological picture of pulmonary oedema and was therefore admitted to the critical care unit where he was stabilized using high-flow oxygen therapy. At this stage, a trans-thoracic echocardiogram revealed severe AR, a bicuspid aortic valve with normal root and ascending aorta dimensions, anterior mitral valve leaflet aneurysm, severe mitral regurgitation, and good biventricular function (*Figure 2A* and *B*). Multiple site blood cultures grew *S. sanguinis.* The trans-oesophageal echocardiogram confirmed multiple vegetations on the aortic valve causing a perforation in the posterior aortic valve cusp with severe AR and features suggestive of an aortic root abscess. There was a perforation through the anterior mitral valve leaflet with a large aneurysmal segment and a broad jet of mitral regurgitation through it. The diagnosis of left-sided IE was established according to the Duke criteria,^4^ which was later modified with 80% diagnostic accuracy.^5^ An IV amoxicillin 2 g 4 hourly was commenced based on sensitivity results. A CT of thorax, abdomen, and pelvis showed multifocal pyelonephritis primarily on the right. His electrocardiogram demonstrated sinus tachycardia with a PR interval of 153 ms.

The multi-disciplinary team considered the clinical presentation, the presence of acute severe AR, and the recent coiling of the aneurysm and recommended urgent surgery due to the incidence of heart failure and features of uncontrolled infection (suspected root abscess) that are both Class I recommendations for urgent surgery as per current European Society of Cardiology (ESC) guidelines.^6^ The plan was discussed with the patient, and mechanical aortic and mitral valve replacements were decided given the patient’s age. The risk of cerebral haemorrhage from either anticoagulation for cardiopulmonary bypass or subsequently with warfarin was deemed to be low given the prior coiling and 2023 ECS guidelines that support indication for urgent surgery if intracranial haemorrhage volume is <30 mL.^6^

**Figure 3:**
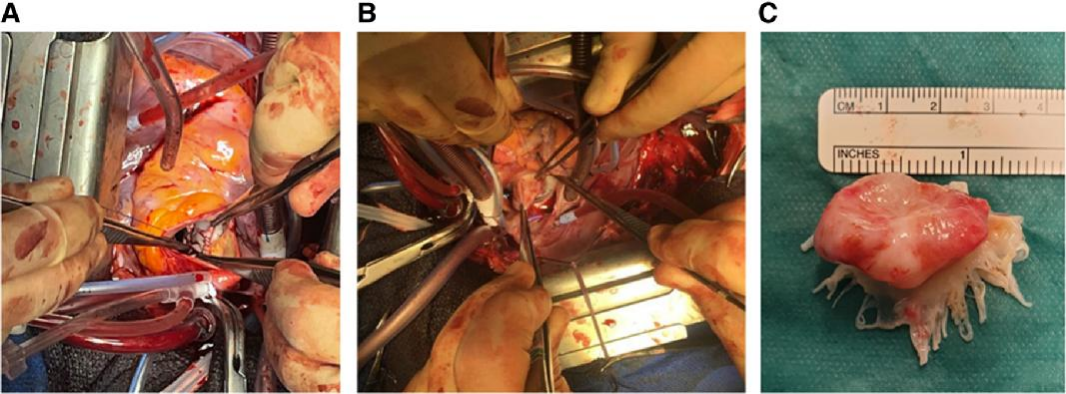
(*A*) The forceps of the surgeon are pointing to the pericardial patch at the aorto-mitral curtain; the view is through the aortic root. (*B*) The surgeon forceps are holding and pointing to the anterior mitral leaflet aneurysm prior excision; the view is trans-septal from the right atrium into the left atrium. (*C*) The excised anterior mitral leaflet with mushroom shape aneurysm bulging from the top (atrial surface).

The patient was taken to theatre 10 days after the presentation for mechanical aortic and mitral valve replacement with patch reconstruction of the aorto-mitral curtain. Intra-operatively, there was a bicuspid aortic valve with multiple small (less than 1 cm) vegetations, interestingly, an endothelialized perforation in the left ventricular outflow tract (LVOT) connecting it to the left atrium (presumably a healed abscess), and a large and broad-based mitral valve aneurysm involving the anterior mitral leaflet with vegetations and perforation (*Figure 3B*). The aneurysm was excised with the anterior mitral valve leaflet (*Figure 3C*), and the LVOT perforation was dealt with using a pericardial patch (*Figure 3A*). Size 23 and 31 mm bicarbon mechanical aortic and mitral valves were implanted, respectively. The patient was anticoagulated initially with additional intravenous heparin until a therapeutic international normalised ratio of 2.5 was reached with warfarin. The patient had a satisfactory post-operative recovery, apart from a short episode of self-terminating atrial fibrillation, before being transferred back to the cardiology ward on Day 4 post-operatively for ongoing antibiotic therapy. Both the intra-operative aortic and mitral valve tissue samples were negative for any microorganism, as were post-operative blood cultures, which hopefully indicated the appropriateness and success of the initial antibiotic regimen. This patient was subsequently discharged home after 6 weeks of antibiotics and has now returned to his previous level of function including returning to work.

## Discussion

This case represents a rare triad syndromic presentation of IE in the form of SAH, mycotic cerebral aneurysm, and mitral valve aneurysm due to left-sided aortic and mitral valve endocarditis. This patient had two risk factors: the bicuspid aortic valve and dental treatments a few months prior to his symptoms. There are 112 cases in the literature, between 1957 and 2022, presenting with SAH, mycotic aneurysms, or mitral valve aneurysm separately or in combination but not all three simultaneously. Hence, to our knowledge, this is the first case of this triad of conditions as a presentation of IE to be reported.

The incidence of SAH in IE ranges 9–12.9% and with concomitant mycotic aneurysm of 4–9%,^7^ with frontal and parietal lobes being the most common sites. The majority of SAH result from secondary haemorrhage after embolic ischaemia.^7,8^ Although ruptured cerebral mycotic aneurysm can be devastating, some may be clinically silent.^9^ A challenge of these cases is the optimum timing for surgery after mycotic SAH. These mycotic aneurysms tend to have a predilection for the peripheral part of the cerebral circulation. Showering of septic emboli leading to mycotic aneurysms or pyogenic arteritis rather than bacteraemia itself is the most likely mechanisms.^10^

Most mitral valve aneurysms involve the anterior mitral leaflet and rarely the posterior leaflet.^3,11–13^ The anterior mitral valve leaflet aneurysm is almost always associated with aortic valve endocarditis due to either the regurgitant jet that strikes the ventricular surface of the anterior mitral leaflet or the direct extension of the infection into the aorto-mitral curtain and mitral valve.^3^ The mitral valve aneurysm has a potential for life-threatening complications such as rupture leading to acute heart failure, fistula formation, or embolization.^12^

The main pitfall in the management of this case was the use of vasopressors to target higher-than-normal blood pressure, increasing the afterload, worsening the AR, and leading to acute heart failure and pulmonary oedema, due to a failure to consider IE as the cause of the symptoms. In severe AR, ideally the afterload would have been reduced instead.

Additionally, the timing of surgery in such cases can be controversial, balancing the risk of further SAH with systemic heparinization vs. early intervention for heart failure. We believe that it is safe to operate on such patients within 10 days of SAH, provided there has been successful endovascular treatment of the mycotic cerebral aneurysm.

In summary, this case scenario highlights several learning outcomes including the importance of holistic clinical examination of the cardiovascular system in a patient presenting with haemorrhagic stroke; a high index of suspicion of IE is required in cases of cerebral aneurysm with no risk factors, especially with a non-specific history of lethargy or flu-like illness; vasopressors are best avoided in the context of AR due to the risk of acute decompensation; finally, cardiac surgery might be safe if performed as early as 10 days after SAH provided that the aneurysm is successfully treated.

## Data Availability

The data underlying this article will be shared on reasonable request to the corresponding author.

## Lead author biography

Ahmed A. M. Abbas is currently a cardiac surgeon in Alkafeel Specialty Hospital in Iraq and Head of medical Education Unit at the University of Warith Aanbiyaa (Karbala-Iraq).He trained in cardiothoracic suergery in the UK in a well knwon Cardiothoracic units (Papworth, Blackpool, Leicester and Middlesbrough) and has a special interest in aortic surgery. He originally graduated from University of Babylon and perused cardiothoracic surgery Fellowship training in the UK and completed the FRCS-CTh.

**Consent:** The authors confirm that written consent was obtained from the patient for the submission and publication of this case, including images, in line with COPE guidance.

**Funding:** None declared.
